# Supplementary material for: Physiological mechanisms of muscle strength and power are dependent on the years post obtaining peak height velocity in elite juniors rowers: A cross-sectional study
Source: PLoS One. 2023 Jun 7;18(6):e0286687. doi: 10.1371/journal.pone.0286687 (PMC10246840; doi:10.1371/journal.pone.0286687)
Supplement: S1 File — (PDF) [file pone.0286687.s001.pdf]

Supplementary file 1

Specifications of equipment used in muscle strength tests

For the upper limb strength test, the exercise bent row on the bench was chosen. For this, a "handmade table" 200 cm long by 29 cm wide and 94 cm high was used (see Figure 1). In addition, an Olympic bar (Unknown brand ®, 20 Kg, 220 cm long, 28mm shaft diameter) and rubberized Olympic washers (Unknown brand ®) were used. The participant started the test lying on the table, in prone position and with the upper limbs extended. The bar with washers was located below the table and aligned with the participant's pectoral. After inserting the initial load, the evaluator of the Brazilian Rowing Confederation (CBR) instructed the participant to hold the bar with a pronated grip. Then, the participant was instructed to pull the bar toward his pectoral.

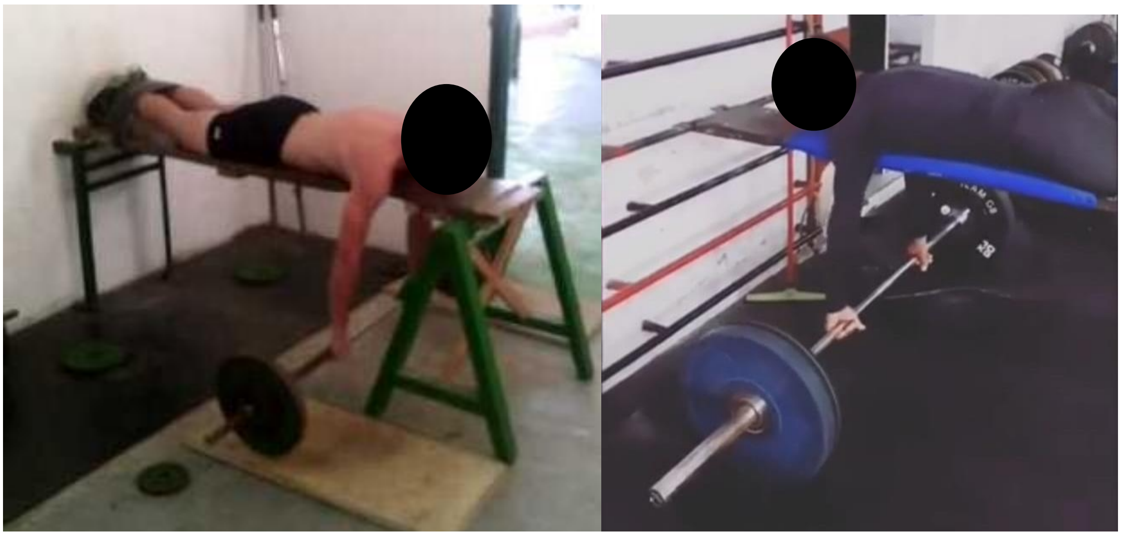

Figure 1. Bent row on the bench.

For the second upper limb strength test, the bench press exercise was chosen. For this, a specific bench was used (Unknown brand ®, 40 Kg, 150 cm long, 122 cm wide and 45 cm high) with bar support (see Figure 2). In addition, an Olympic bar (Unknown brand ®, 20 kg, 220 cm long, 28 mm shaft diameter) and rubberized Olympic washers (Unknown brand ®) were used. The participant was instructed by the CBR evaluator to lie down, in dorsal decubitus, on the specific bench to perform the supine press. After positioning himself, the participant was instructed to grip the bar using the pronated grip so that his arms were at a 90° angle. Next, the participant was instructed to extend his arms to remove the bar from the support and to lower the bar until it touched his pectoral.

After this, the participant was instructed to extend his arms again and place the bar on the support.

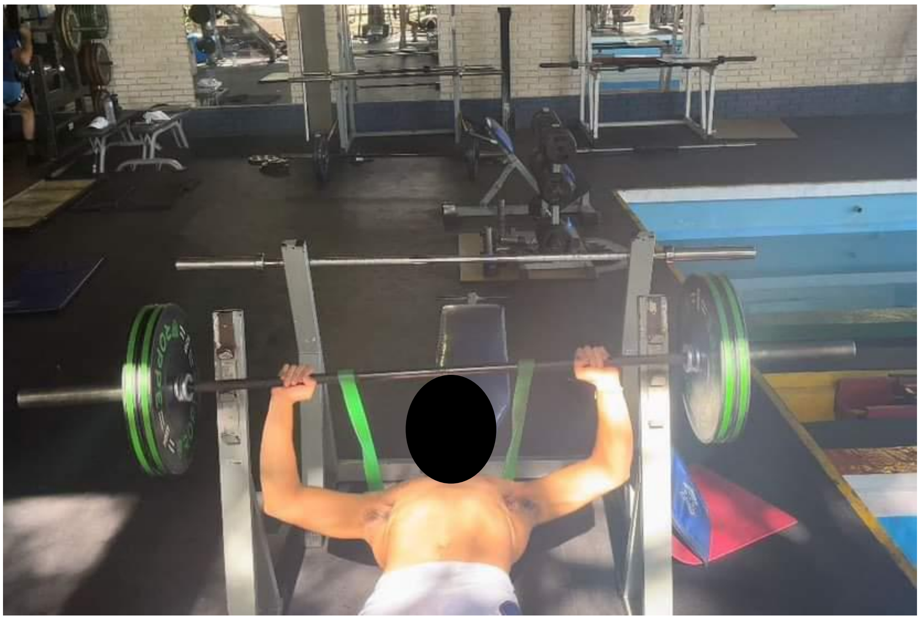

Figure 2. Bench press.

For the lower limb strength test, the squat exercise was chosen. For this, we used a 134.6 cm long (ground base), 121.3 cm wide and 172.6 cm high rack (See Figure 3). An Olympic bar (Unknown brand, 20 kg, 220 cm long, 28 mm shaft diameter) and rubberized Olympic washers (Unknown brand) were used. After the initial load insertion, the CBR evaluator instructed the participant to position himself under the bar and to perform the grip in pronated position so that his arms were at a 90° angulation. Subsequently, the participant was instructed to "pull out" the bar and perform a squat until his knees reached a 90° angulation.

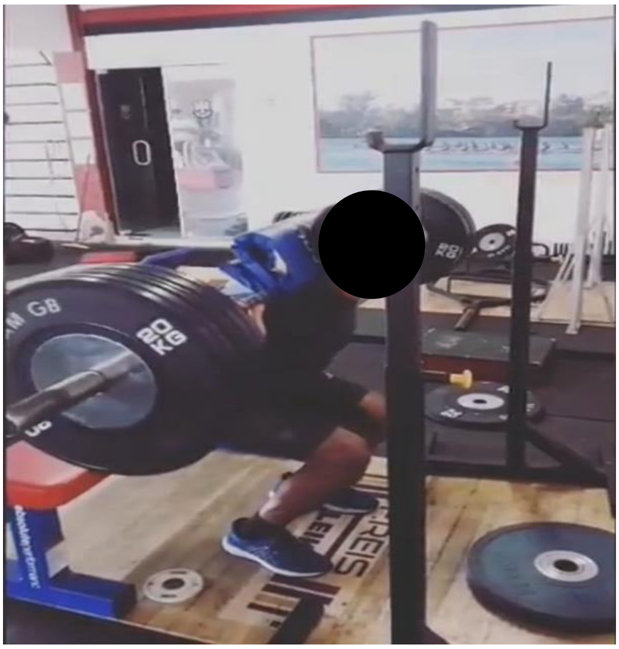

Figure 3. Squat.

For the second lower limb strength test, the deadlift exercise was chosen (See Figure 4). An Olympic bar (Unknown brand ®, 20 Kg, 220 cm long, 28mm shaft diameter) and rubberized Olympic washers (Unknown brand ®) were used. The bar with washers was positioned on the ground. After the insertion of the initial load, the CBR evaluator instructed the participant to position with the legs against the bar, bend the knees and perform the grip in pronated position with arms extended. Subsequently, the participant was instructed to perform a knee and hip extension, lifting the load from the ground.

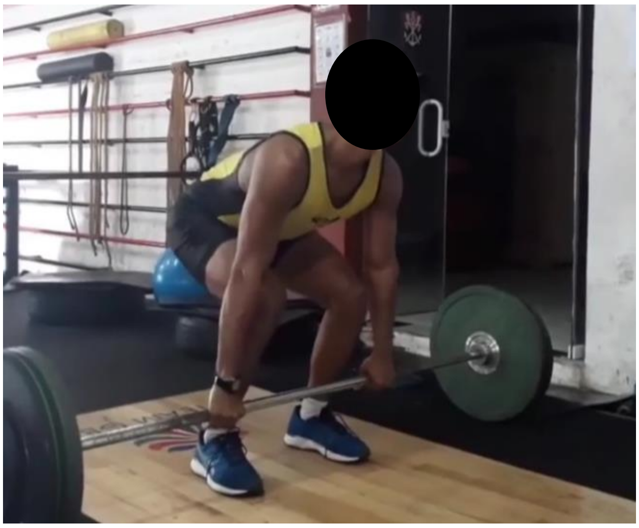

Figure 4. Deadlift.
