# Supplementary material for: Physiological mechanisms of muscle strength and power are dependent on the years post obtaining peak height velocity in elite juniors rowers: A cross-sectional study
Source: PLoS One. 2023 Jun 7;18(6):e0286687. doi: 10.1371/journal.pone.0286687 (PMC10246840; doi:10.1371/journal.pone.0286687)
Supplement: S2 File — (DOCX) [file pone.0286687.s002.docx]

**Supplementary file 2**

**Table s-1**. Standard error of the performance measures. s-1: Supplementary Table 1.

| Variables | | Male sex _(n = 171)_ | | | Female sex _(n = 64)_ | | |
| --- | --- | --- | --- | --- | --- | --- | --- |
|  | YPPHV groups: | Recent | Median | Veteran | Recent | Median | Veteran |
| 100-m _(sec)_ | | 1.1 | 1.9 | 0.1 | 6.8 | 0.2 | 0.2 |
| 500-m _(sec)_ | | 0.9 | 0.7 | 1.3 | 1.4 | 1.3 | 1.4 |
| 2,000-m _(min)_ | | 0.0 | 0.1 | 0.1 | 0.1 | 0.1 | 0.1 |
| 6,000-m _(min)_ | | 0.3 | 0.2 | 0.2 | 0.5 | 0.5 | 0.6 |
| 100-m _(Watts)_ | | 13.3 | 13.2 | 15.4 | 11.3 | 10.1 | 17.9 |
| 100-m _(Watts/Kg)_ | | 0.2 | 0.1 | 0.1 | 0.1 | 0.2 | 0.2 |
| 500-m _(Watts)_ | | 8.9 | 8.6 | 8.6 | 15.0 | 17.6 | 17.4 |
| 500-m _(Watts/Kg)_ | | 0.1 | 0.1 | 0.0 | 0.2 | 0.2 | 0.2 |
| 2,000-m _(Watts)_ | | 8.1 | 8.5 | 10.2 | 16.9 | 15.2 | 17.3 |
| 2,000 _(Watts/Kg)_ | | 0.0 | 0.1 | 0.1 | 0.2 | 0.2 | 0.2 |
| 6,000-m _(Watts)_ | | 9.4 | 9.5 | 11.4 | 15.5 | 15.8 | 19.2 |
| 6,000-m _(Watts/Kg)_ | | 0.1 | 0.1 | 0.1 | 0.2 | 0.1 | 0.2 |
| 1RM in Bench press _(Kg)_ | | 1.9 | 2.0 | 2.0 | 3.5 | 1.4 | 4.1 |
| 1RM in Rowing lyving down _(Kg)_ | | 1.6 | 1.8 | 1.7 | 2.9 | 1.6 | 3.5 |
| 1RM in Squat _(Kg)_ | | 2.6 | 3.0 | 3.1 | 4.0 | 2.8 | 8.1 |
| 1RM in Deadlift _(Kg)_ | | 3.6 | 4.2 | 3.4 | 7.8 | 3.3 | 8.9 |

n: Absolut number. YPPHV: Years post peak height velocity. Sec: Second’s. -m: meters. Min: Minutes. Kg: kilograms. 1RM: One repetition maximum.

Table s-1 shows the standard error values of the variables of indoor rowing performance and one repetition maximum (1RM) strength tests.
