# Supplementary material for: Physiological mechanisms of muscle strength and power are dependent on the years post obtaining peak height velocity in elite juniors rowers: A cross-sectional study
Source: PLoS One. 2023 Jun 7;18(6):e0286687. doi: 10.1371/journal.pone.0286687 (PMC10246840; doi:10.1371/journal.pone.0286687)
Supplement: S3 File — (PDF) [file pone.0286687.s003.pdf]

Physiological mechanisms of muscle strength and power are dependent on the years post obtaining Peak Height Velocity in elite Juniors rowers

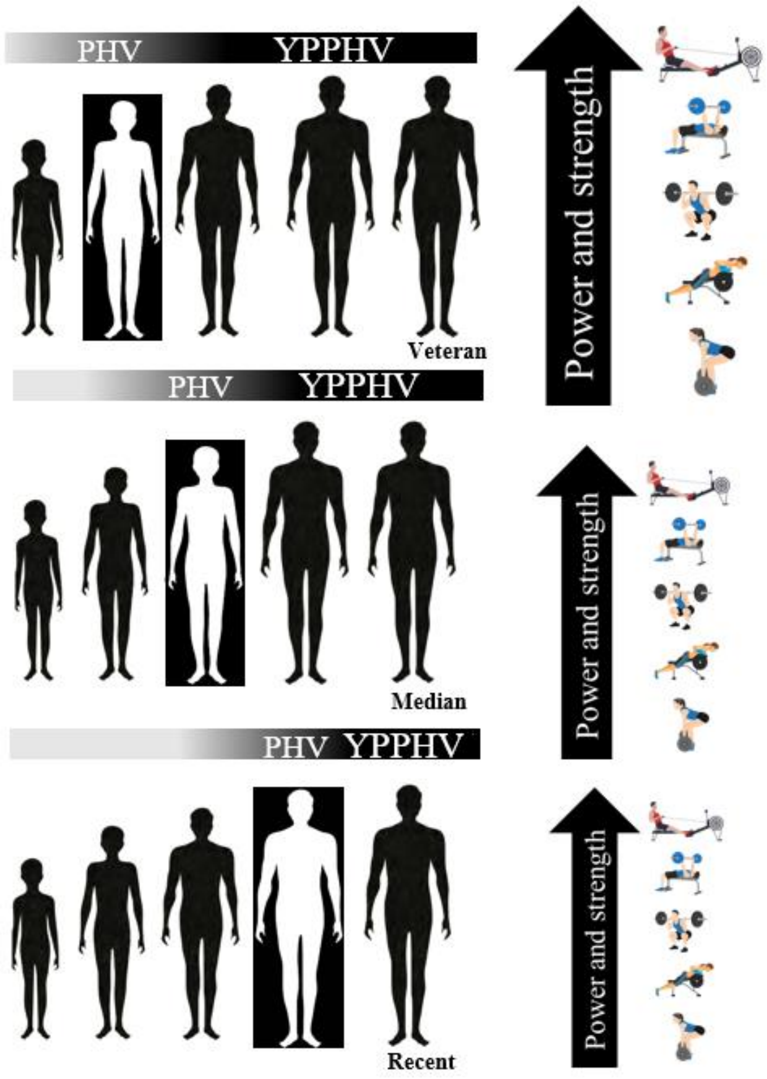

Key-Points

- In male athletes, muscle power produced during tests performed in indoor rowing and muscle strength were superior in YPPHV-veteran athletes compared to their recent and median YPPHV peers.

- In female athletes, test time and muscle power produced during 500-m test performed in indoor rowing were superior in YPPHV-veteran athletes compared to their recent and median YAPHV peers.

- Apparently the residual effects of early maturation in relation to the power and muscle strength advantage still persist until the age of 18 in rowers of both sexes.

Legend: PHV: Peak height velocity. YPPHV: years post PHV

PLOS ONE
