# Supplementary material for: Physiological mechanisms of muscle strength and power are dependent on the years post obtaining peak height velocity in elite juniors rowers: A cross-sectional study
Source: PLoS One. 2023 Jun 7;18(6):e0286687. doi: 10.1371/journal.pone.0286687 (PMC10246840; doi:10.1371/journal.pone.0286687)
Supplement: Attachment — Submitted filename: PONE-D-23-05680v1.doc [file pone.0286687.s004.doc]

**Model for Manuscript Review**

**Research articles**

**Manuscript title:**

PONE-D-23-05680- Physiological mechanisms of muscle strength and power are dependent on the years post obtaining Peak Height Velocity in elite Juniors rowers?

| **Títle** |  |
| --- | --- |
| Is it understandable and concise? | ( x ) Yes ( ) Not |
| Reflects the content? | ( x ) Yes ( ) Not |
| **Abstract** |  |
| It includes: objectives, methodology, key findings and conclusions? | ( x ) Yes ( ) Not |
| **Introduccion** |  |
| The investigation was carried out in a suitable theoretical structure? | ( x ) Yes ( ) Not |
| Clear leaves the questions you want to answer and objectives of the work? | ( ) Yes ( x ) Not |
| The cited references are current and relevant? | ( x ) Yes ( ) Not |
| **Methods** |  |
| The methods presented are appropriate to achieve the proposed objectives? | ( x ) Yes ( ) Not |
| The selection and composition of the sample are adequately described? | ( ) Yes ( x ) Not |
| The data collection process and the tools used are described clearly? | ( x ) Yes ( ) Not |
| The statistical analysis and the research design appropriate? | ( x ) Yes ( ) Not |
| **Results** |  |
| The presentation of the results clear? | ( x ) Yes ( ) Not |
| The main results are highlighted without the inclusion of interpretation and comparisons? | ( x ) Yes ( ) Not |
| The results evaluate the proposed objectives? | ( x ) Yes ( ) Not |
| Tables and figures are properly numbered, labeled and explained? | ( x ) Yes ( ) Not |
| **Discussion and Conclusion** |  |
| The results are discussed based on the literature? | ( x ) Yes ( ) Not |
| Author's interpretations show the safety and soundness? | ( x ) Yes ( ) Not |
| The limitations of the work are presented? | ( x ) Yes ( ) Not |
| The conclusions of the study are presented? | ( x ) Yes ( ) Not |
| The conclusions respond to the objectives? | ( ) Yes ( x ) Not |

**General comments:**

**Title**

Are presented satisfactorily.

**Abstract**

Are presented satisfactorily. I suggest adding some absolute and statistical results to help visualize the work.

Please confirm that the keywords appear as descriptors in health sciences.

**Introduction**

It should initially present a more general approach, gradually address the problem (gap), and then present the objective. The problem must be better identified. Mentioning that PHV and its relationship to what the study proposes is still unclear would not be a problem. I suggest continuing with the statements for and against the aforementioned so that we can naturally trigger the objectives.

**Methods**

It should present the design of the study. A CONSORT or timeline should be presented in order to get a better view of the study design.

The sample should be better explained with the number of subjects presented initially and then present the inclusion and exclusion criteria. As it was determined that 235 junior rowers (171 men and 64 women) would be needed or sufficient. please clarify.

**Results**

Are presented satisfactorily.

**Discussion**

Are presented satisfactorily.

**Conclusion**

Are presented satisfactorily. However, it would be necessary to present the practical applications of the results found.

**References**

Are presented satisfactorily. However, of the 45 references, only 15 are current and 30 have more than five years of publication. Please update the theoretical framework.

**Overview**

The manuscript presented addresses a relevant research topic.

It would be advisable to do a general review.

**Specific comments and suggestions:**

**Outcome evaluation**

- Accept unchanged	(	)
- Accepted with minor changes	( x )
- Accepted with major changes	(	)
- Rejected	(	)
