# Supplementary material for: Physiological mechanisms of muscle strength and power are dependent on the years post obtaining peak height velocity in elite juniors rowers: A cross-sectional study
Source: PLoS One. 2023 Jun 7;18(6):e0286687. doi: 10.1371/journal.pone.0286687 (PMC10246840; doi:10.1371/journal.pone.0286687)
Supplement: Attachment — Submitted filename: R2 - Responses to reviews PONE-D-23-05680v1.doc [file pone.0286687.s006.doc]

**Response to reviews**

**Manuscript title:**

PONE-D-23-05680- Physiological mechanisms of muscle strength and power are dependent on the years post obtaining Peak Height Velocity in elite Juniors rowers?

**Reviewer #1**:

Thank you for this revision. All of the points I raised have been adequately addressed. However, it is now the case that when the results are considered relative to mass, only the differences for 100m performance are relevant. Even when viewed in absolute terms, there is significant overlap in the results, including in the 100m test performances. This raises the question of why trainers and coaches need to consider peak height velocity/maturation at all. The results seem to show a strong overlap, suggesting that these factors may not be as relevant as previously thought.

Overall, the interpretation of the results and the discussion should focus more on the implications of these findings for trainers and athletes. Why is it not sufficient to simply consider biological age? Combining biological age and mass, for example, could already explain a large portion of the variance in performance. These aspects should definitely be added or highlighted more prominently in the revised version.

**Answer**: In the results section, in the description of Table 1, we address the difference in body weight between the groups (lines 256 to 258). In addition, in the discussion session we inserted a topic on “Biological maturation, body weight and performance in rowers” (lines 384 to 402). Finally, in the concluding section, we gave a suggestion of practical applicability aimed at nutritional intervention to increase body weight in recent-YPPHV athletes (lines 436 to 440).

-------------------------------------------------------------------------------------------

**Reviewer #3**:

Dear authors, congratulations for your work. Your revision has improved the quality of the document and it is now ready for acceptance.

**Answer**: Thanks’.
